# Transcriptomic profile of host response in Japanese encephalitis virus infection

**DOI:** 10.1186/1743-422X-8-92

**Published:** 2011-03-04

**Authors:** Nimesh Gupta, PV Lakshmana Rao

**Affiliations:** 1 Division of Virology, Defence Research and Development Establishment, Jhansi Road, Gwalior-474002, India

## Abstract

**Background:**

Japanese encephalitis (JE) is one of the leading causes of acute encephalopathy with the highest mortality rate of 30-50%. The purpose of this study was to understand complex biological processes of host response during the progression of the disease. Virus was subcutaneously administered in mice and brain was used for whole genome expression profiling by cDNA microarray.

**Results:**

The comparison between viral replication efficiency and disease progression confirms the active role of host response in immunopathology and disease severity. The histopathological analysis confirms the severe damage in the brain in a time dependent manner. Interestingly, the transcription profile reveals significant and differential expression of various pattern recognition receptors, chemotactic genes and the activation of inflammasome. The increased leukocyte infiltration and aggravated CNS inflammation may be the cause of disease severity.

**Conclusion:**

This is the first report that provides a detailed picture of the host transcriptional response in a natural route of exposure and opens up new avenues for potential therapeutic and prophylactic strategies against Japanese encephalitis virus.

## Background

The host response to infection is central to the effective control and ultimate clearance of invading pathogens or removal of infected cells. Infection of host with a viral pathogen marks the onset of changes in the host cell's microenvironment. Such changes in host gene expression could be a cellular antivirus response, a virus induced response that facilitate its own replication and spread or a non-specific response that neither promotes nor prevents virus infection. This alteration of expression of many cellular genes can be identified using cDNA microarray [1].

Defining the transcriptional regulation of host genes on virus infection can be used as a tool to obtain an elaborate insight into mechanisms of host-virus interactions and to unravel the molecular basis of disease pathogenesis. Viruses from several families can infect neurons in the CNS (Central Nervous System) and the study of gene expression changes in the CNS during virus infection can lead to identification of new genes whose function is essential either for the promotion or prevention of virus infection [2,3].

Japanese Encephalitis is one of the most dreaded mosquito borne encephalitis virus causing acute encephalitis in humans. Among the medically important flaviviruses, JEV infection has the highest mortality rate of 30-50% [4,5] and remains as a major public health problem in several parts of Asia. The main concern is the constant spreading of JE to new geographical areas [6]. Better understanding of JEV pathogenesis is required to identify risk factors for progression of disease and viral persistence, which may help in the development of differential diagnostics and new therapeutic interventions.

In a previous study, employing cDNA microarray, we identified various antiviral genes along with the innate immune response related chemokine expression at very early phase of infection in mouse neuronal cells [7]. However, there is no information available on genome wide host gene expression changes induced by JEV in the CNS and in a natural route of infection. There is a requirement to understand the molecular events responsible for disease progression, viral persistence and complex biological processes of host response during the complete course of JEV infection, starting from peripheral route to CNS, neuroinflammation, disease severity and death.

Thus, we employed cDNA microarray for the systematic analysis of global host transcriptional responses in CNS of JEV infected mice. In the recent epidemics, it has been observed that the mortality rate in JEV infected patients is higher in 1-5 years of age group with immature immune system. So we employed a young mice model to explore the precise molecular events involved in JEV infection of CNS during the disease severity. Subcutaneous challenge of JEV in one-week-old mice provides a natural route of exposure to study molecular mechanism of JEV pathogenesis in CNS. In our earlier report using this animal model we observed a significant regulation of IFN-γ in virus infected mice spleen, which demonstrates a specific but insufficient anti-viral response in the periphery to limit virus spread to brain [8]. Thus this model with immature immune system may provide important information about the role of host response in disease severity. The reproducibility of the infection and diseases symptoms was verified with a significant number of experimental repetitions in newly established animal model and there was no variation in the disease symptoms of the individual animals at any time point of infection and the mortality rate was also 100% at 6 DPI (Day post-infection).

It has already been reported that cerebral cortex is an important site of virus replication, inflammation, injury and is associated with encephalitis in the JEV-infected host [9,10]. Thus we employed brain cerebral cortex for the investigation of transcriptomic profile of host response in JEV infection. Our results thus provide a genome-wide investigation of an animal model of JEV infection and a genomic view of systemic host-virus interactions during infection.

## Results

### Evaluation of viral load and histopathological analysis in mice CNS after subcutaneous infection with JEV

**Figure 1:**
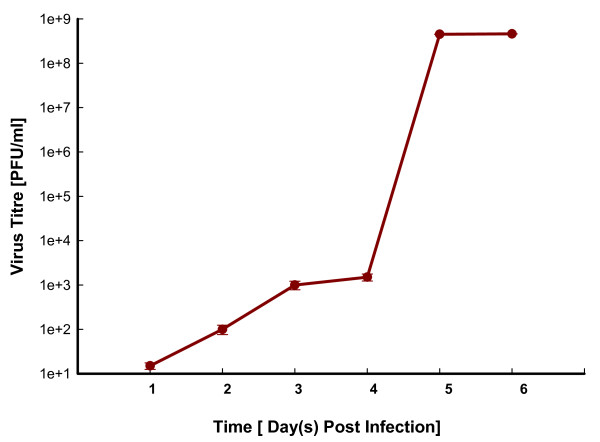
**Viral load in mouse brain infected with JEV**. Viral load in the cerebral cortex of mice infected with 10^3 ^PFU of JEV at various days post-infection (DPI). Infectious virus titre was determined using Plaque formation assay on porcine stable (PS) kidney cells. Values are mean ± SE of four replicates each.

Animals showed JE specific symptoms with the progression in severity in a time dependent manner. After subcutaneous inoculation, virus load in brain was evaluated up to 6 DPI by plaque titration assay (Figure 1). JEV replicates rapidly in brain with an increase in titre from 2 DPI and reached a maximal viral load at 5 DPI. Subsequently, the viral load remained constant until death at 6 DPI. Figure S1 (Additional file 1) is showing the histopathological analysis of cerebral cortex from mock- and JEV-infected animals with the pathological changes after viral infection. The analysis showed congestion and dilation of blood vessels at 1 DPI. A time dependent increase in mononuclear cell infiltration, prominent peri-vascular cuffing and neuronal degeneration was observed in further time points of infection.

### Host transcription profile after JEV infection

**Figure 2:**
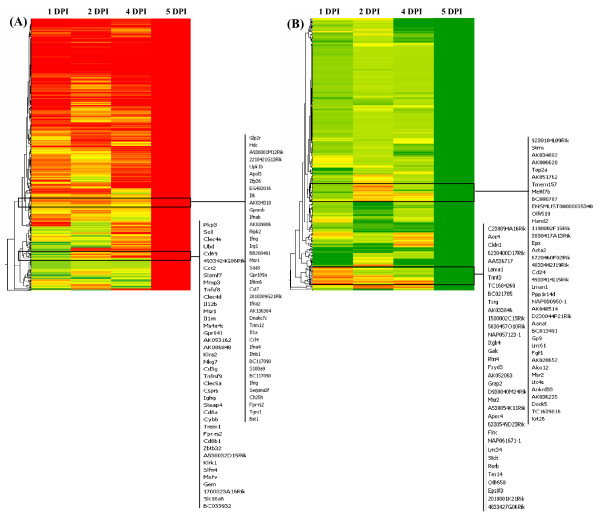
**Global gene expression profiles in the brain of BALB/c mice in response to JEV infection**. Genes were sorted using hierarchical clustering, average linkage and Pearson uncentred correlation. Green and red bands represent downregulated or upregulated levels of mRNA expression relative to mock-infected mouse, respectively. (A) Genes upregulated (≥2.0-fold changes) with P ≤ 0.05. (B) Genes downregulated (≤ -2.0-fold change) with P ≤ 0.05.

**Figure 3:**
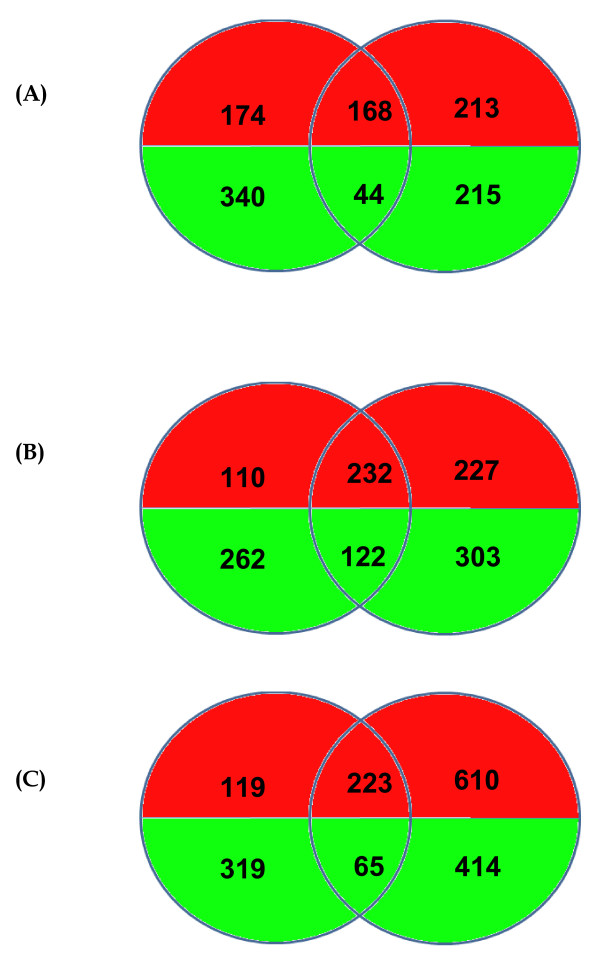
**Comparison of gene expression at different days of infection**. A Venn diagram depicting the differentially expressed genes (≥2.0-fold change or ≤ -2.0-fold change; P ≤ 0.05) unique or common at (A) 1 DPI versus 2 DPI (B) 1 DPI versus 4 DPI and (C) 1 DPI versus 5 DPI. Left circle corresponds to the 1 DPI while right circle in each figure corresponds to 2, 4 and 5 DPI respectively. Green and red indicates down regulated and up regulated genes respectively.

Using whole genome microarray the host transcription profile was studied in mouse brain, the data from mock- and JEV-infected animals were compared in time course study after 1, 2, 4 and 5 DPI. Overall, the largest difference in transcription levels between the mock- and JEV-infected groups was detected at 5 DPI, corresponding to the time of maximum viral load in brain. A total 833 genes were up regulated and 479 genes were down regulated at 5 DPI (Table 1). The hierarchical clustering also showed significant regulation of a large set of genes at 5 DPI (Figure 2a and 2b). Gene expression profile at 1 DPI was compared with 5 DPI and 288 genes showed similar regulation in their expression. However, the more similarity in gene expression was observed when 1 DPI was compared with 4 DPI (Figure 3). The gene modulation seems to correlate with viral replication and classification of genes reveals critical role of inflammation and immune response in JEV infection (Table 2).

**Table 1:** Differentially expressed genes in mouse brain at different days after JEV infection.

Time [Day post infection]	Up	Down
Day 1	342	384
Day 2	381	259
Day 4	459	425
Day 5	833	479

**Table 2:** Representation of statistically significant genes categorised into genetic pathways by the KEGG classification system, in mouse brain after JEV infection.

KEGG Pathway	Number of genes	P-value
Intracellular signaling cascade	192	1.4E-05
Negative regulation of biological process	187	0.0002
Immune response	136	1.7E-25
Cell adhesion	132	1.03E-05
Defense response	106	1.2E-09
Apoptosis	77	0.001
Inflammatory response	44	1.8E-05
Chemotaxis	39	6.2E-08
Antigen processing and presentation	39	4.3E-16
Integrin-mediated signaling pathway	26	0.0008
G-protein-coupled receptor binding	19	0.0001
Interleukin binding	18	2.1E-05
Response to virus	18	3.1E-05
Cytokine and chemokine mediated signaling pathway	17	0.0001
Chemokine activity	16	3.3E-05
Chemokine receptor binding	16	3.3E-05
Complement activation	14	0.0003
Leukocyte mediated immunity	11	0.0005
MHC protein complex	10	1.6E-08
Immunoglobulin mediated immune response	10	0.0004
Leukocyte chemotaxis	10	0.001

### Expression profile of cell surface molecules in brain after JEV infection

The host transcription profile in brain showed the effect of JEV infection on expression of various cellular receptors. Gene ontology analysis of these significantly activated cell surface receptors showed their involvement in immune response, inflammatory response, cell adhesion, defense response, proteolysis, endocytosis and chemokine signaling (Table 3). The lectin receptors like Clec7a, Clec5a, Clec4e involved in the immune responses showed a high level of expression at 5 DPI along with other receptors involved in immune response like Masp2. Receptors involved in immune response like Cd8a and Cd40 were significantly up-regulated at 5 DPI only except Lgals3bp which has showed significant up regulation at 1 DPI also. The receptor involved in inflammatory response showed high level of expression at 5 DPI like Tlr (Toll-like receptor) 2, Tlr7, Edg3, Tnfrsf1b and Cd14 except sialic acid receptor Siglec1 and Tlr4 which showed significant regulation at 1 DPI also (Table 3). The receptors of cell adhesion family like Sell, Icam1, Amica1, Vcam1, Itgb2 and Itgam showed high level of expression at 5 DPI. The receptors involved in signal transduction like Gpr84 showed increase in their expression in association with the disease progression. However, receptors like Osmr and Admr were significantly up regulated at 5 DPI except Ccrl2 which was up regulated at 2 DPI. The receptors involved in defense response showed varying levels of expression, like Tlr1 was up regulated throughout the disease course with increase in up regulation at 5 DPI while C180 showed a gradual increase in regulation with significant up regulation at 4 and 5 DPI. Another receptor Clec2 d, involved in defense response was up regulated at 1 DPI and further up regulation was found at 4 and 5 DPI. Interestingly, Tlr3 showed up regulation at all the time points of infection but there was an increase in level of expression at 1 and 4 DPI. However, T cell mediated cytotoxicity receptor Ptprc showed high level of expression at 4 and 5 DPI. Interestingly, receptors involved in cytokine and chemokine signalling like Csf2rb2, Csf2rb1, Ccr5 and Il2rb showed significant up regulation only at 5 DPI except Lilrb3 which was significantly up regulated at 1 DPI also (Table 3). Various other receptors like Msr1, Fas, Selp, Traf1, Treml4 and Pik3ap1 also showed significant up regulation at 5 DPI.

**Table 3:** Differential expression of cell surface molecules in brain after JEV infection.

Biological Process	Gene Symbol	Accession No	Description	Fold change over mock-infected			
				
				1 DPI	2 DPI	4 DPI	5 DPI
Immune response	Clec7a†	NM_020008	C-type lectin domain family 7	0.49	2.34	1.32	6.34
Immune response	Clec5a	NM_001038604	C-type lectin domain family 5	1.32	1.85	2.07	5.90
Protein binding scavenger	Lgals3bp	NM_011150	Lectin, galactoside-binding, soluble, 3 binding protein	4.23	3.58	3.92	4.90
Inflammatory response	Tlr2†	NM_011905	Toll-like receptor 2	0.87	1.34	1.40	4.90
Cell adhesion	Sell	NM_011346	Selectin, lymphocyte	-0.96	-0.55	-0.60	4.85
Inflammatory response	Siglec1†	NM_011426	Sialic acid binding Ig-like lectin 1	2.39	2.80	2.29	4.83
Immune response	Clec4e	NM_019948	C-type lectin domain family 4	-0.87	-0.46	-0.61	4.57
Signal transduction	Gpr84	NM_030720	G protein-coupled receptor 84	2.10	2.66	3.25	4.47
Endocytosis	Msr1	NM_031195	Macrophage scavenger receptor 1	-0.94	-0.56	1.10	4.40
Defense response	Tlr3†	NM_126166	Toll-like receptor 3	3.56	2.47	5.03	4.27
T cell mediated cytotoxicity	Ptprc	NM_011210	Protein tyrosine phosphatase, receptor	1.63	1.78	2.00	4.27
Signal transduction	Ccrl2†	NM_017466	Chemokine (C-C motif) receptor-like 2	1.66	2.50	1.98	4.16
Cytokine & Chemokine signaling	Lilrb3	NM_011095	Leukocyte immunoglobulin-like receptor	2.06	1.94	0.65	4.09
Immune response	Lgals3	NM_010705	Lectin, galactose binding, soluble 3	1.58	1.40	1.40	4.00
Defense response	Tlr1	NM_030682	Toll-like receptor 1	2.38	2.15	2.34	3.92
Apoptosis	Fas	NM_007987	Fas (TNF receptor superfamily)	0.24	0.79	0.77	3.90
Antibody-dependent cellular cytotoxicity	Fcgr1	BC025535	Fc receptor, IgG, high affinity I	4.23	1.72	3.05	3.79
Immune response	Masp2†	NM_010767	Mannan-binding lectin serine peptidase	1.66	2.74	3.70	3.75
ADP-ribosylation	Plec1	BC024074	Plectin 1	2.35	1.15	2.57	3.71
Inflammatory response	Tlr4	NM_021297	Toll-like receptor 4	2.11	1.96	2.15	3.57
Cytokine & Chemokine signaling	Csf2rb2	NM_007781	Colony stimulating factor 2 receptor	1.10	1.08	1.38	3.57
Inflammatory response	Selp	NM_011347	Selectin, platelet	-0.06	0.54	-0.67	3.57
Cell adhesion	Icam1†	BC008626	Intercellular adhesion molecule	0.85	0.85	0.82	3.57
NC	Lgals9	NM_010708	Lectin, galactose binding, soluble 9	2.27	1.18	2.41	3.54
Cytokine & Chemokine signaling	Csf2rb1	AK154286	Colony stimulating factor 2 receptor	0.57	1.23	1.43	3.52
Immune response	Il18bp	NM_010531	Interleukin 18 binding protein	2.59	0.90	1.72	3.32
NC	Fcgr4	NM_144559	Fc fragment of IgG, low affinity IIIa, receptor	2.06	1.17	3.39	3.31
Inflammatory response	Tlr7†	NM_133211	Toll-like receptor 7	0.52	0.97	1.80	3.26
Cell adhesion	Amica1	NM_001005421	Adhesion molecule, interacts with CXADR antigen 1	1.08	0.78	0.72	3.24
Immune response	Cd8a	NM_001081110	CD8 antigen, alpha chain	-0.97	-0.55	-0.60	3.15
Cell adhesion	Vcam1	NM_011693	Vascular cell adhesion molecule 1	1.06	0.54	0.83	3.13
Phagocytosis	Fcgr2b	NM_010187	Fc receptor, IgG, low affinity IIb	0.19	0.65	0.17	3.09
Signal transduction	Osmr	NM_011019	Oncostatin M receptor	0.52	0.36	1.19	2.86
NC	Il21r	NM_021887	Interleukin 21 receptor	1.63	1.21	1.04	2.85
Defense response	Cd180	NM_008533	Lymphocyte antigen 78	-1.40	1.32	2.41	2.85
Defense response	Clec2d	NM_053109	C-type lectin domain family 2	2.14	0.97	2.40	2.83
NC	Lair1	NM_178611	Leukocyte-associated Ig-like receptor	1.01	1.48	1.27	2.80
Type IIa hypersensitivity	Fcer1g	NM_010185	Fc receptor, IgE, high affinity I, gamma polypeptide	0.98	1.21	0.81	2.78
Signal transduction	Admr	NM_007412	Adrenomedullin receptor	1.38	0.61	0.84	2.71
Cell adhesion	Itgb2	NM_008404	Integrin beta 2	0.60	1.11	0.58	2.68
Signal transduction	Pilra	NM_153510	Paired immunoglobin-like type 2	1.20	2.17	1.38	2.64
Apoptosis	Traf1	NM_009421	Tnf receptor-associated factor 1	1.12	0.64	0.62	2.62
Signal transduction	Il15ra	NM_008358	Interleukin 15 receptor, alpha chain	1.29	0.77	1.52	2.60
Inflammatory response	Edg3	NM_010101	Sphingolipid g-protein-coupled receptor,	1.66	1.63	1.71	2.57
Signal transduction	Il10ra	NM_008348	Interleukin 10 receptor, alpha	0.72	1.06	0.89	2.55
NC	Paqr5	NM_028748	Progestin and adipoq receptor family member V	1.24	1.49	2.51	2.47
NC	Treml4	NM_172623	Triggering receptor expressed on myeloid cells-like 4	0.33	0.83	-0.69	2.45
Cell adhesion	Itgam	NM_008401	Integrin alpha M	0.66	0.71	1.60	2.44
Proteolysis	Arts1	NM_030711	Type 1 TNF receptor shedding aminopeptidase regulator	1.36	0.98	1.50	2.40
Signal transduction	Pik3ap1	NM_031376	Phosphoinositide-3-kinase adaptor protein 1	0.96	1.25	1.23	2.37
Inflammatory response	Tnfrsf1b	NM_011610	Tumor necrosis factor receptor superfamily, member 1b	0.56	0.47	0.39	2.31
Signal transduction	Il12rb1	NM_008353	Interleukin 12 receptor, beta 1	1.06	0.57	0.35	2.25
Chemotaxis	Ccr5	NM_009917	Chemokine (C-C motif) receptor 5	1.18	1.66	1.40	2.22
NC	Il2rg	NM_013563	Interleukin 2 receptor, gamma chain	1.28	0.43	-0.28	2.19
Inflammatory response	Cd14	NM_009841	Cd14 antigen	0.15	0.78	0.14	2.12
Proteolysis	Lnpep	NM_172827	Leucyl/cystinyl aminopeptidase	0.78	1.01	0.67	2.10
Immune response	Cd40	NM_170701	CD40 antigen	0.67	0.24	0.35	2.10
Cytokine & Chemokine signaling	Il2rb	NM_008368	Interleukin 2 receptor, beta chain	0.17	0.63	0.09	2.10

† Gene regulation was validated by Real time qRT-PCR.

Genes were considered significantly upregulated or downregulated if the change in their relative expression levels was ≥ 2 fold or ≤ -2 fold, respectively.

NC Not classified

### Differential expression of genes in brain after JEV infection

Members of a large subset of genes involved in immune response and inflammation were differentially expressed in brain of JEV-infected animals. Within this group of immune response, expression of Oligoadenylate synthetase family members were significantly upregulated during the disease course except Oas3 which showed gradual increase in its expression from 1 to 5 DPI. The genes belonging to GTPase family also showed very high level of expression during the disease course. Other genes involved in immune response which were up regulated in all time point of infection were Mpa 21, Mx2, Mx1 Phf11, Tgtp, B2 m and Tap1 (Table S1, Additional file 2). The inflammatory response genes include a large number of CXC/CC chemokines family genes (Table S2, Additional file 3). Cxcl10, Ccl12, Cxcl9, Cxcl11, Cxcl13 and Tnf-α showed up regulation at 1 DPI with decline at 2 DPI and further up regulation up to 5 DPI. Ptx3, Ccl3, Spp1, Ccl9, Mefv and Ccl8 showed significant up-regulation at 5 DPI only. The inflammatory genes like Il1β and Il1α showed up regulation from 4 DPI onwards while Il6 was significantly up regulated at 5 DPI only. Other important cluster of genes, which are involved in defense response (Table S3, Additional file 4), anti-viral response of IFN pathway (Table S4, Additional file 5) and genes involved in proteolysis (Table S5, Additional file 6), also showed significant regulation of expression at early or acute phase of disease. The genes, which showed significant down-regulation during the disease course, are involved in cell cycle, endocytosis, leukotriene metabolic response, signal transduction, transcription, cell adhesion and apoptosis (Table 4).

**Table 4:** Down regulated genes expressed in brain after JEV infection.

Biological process	Gene Symbol	Accession No	Description	Fold change over mock-infected			
				
				1 DPI	2 DPI	4 DPI	5 DPI
Defense response	H2-Q1	U96752	Major histocompability complex Q1b	-1.56	-2.45	-4.46	-4.35
Endopeptidase Inhibitor	Pbp2	NM_029595	Phosphatidylethanolamine binding protein 2	-2.67	-4.40	-1.91	-4.28
Cell cycle	Bmpr2	NM_007561	Bone morphogenic protein receptor	-3.94	-4.10	-3.91	-4.02
Oxidative stress	Epx	NM_007946	Eosinophil peroxidase	-1.05	1.05	-1.81	-3.75
Vasoconstriction	Avp	NM_009732	Arginine vasopressin	-3.09	3.04	-2.71	-3.57
T cell mediated cytotoxicity	Ptprc	AK088215	Protein tyrosine phosphatase, receptor type, C	-0.58	-0.55	-0.62	-3.35
Inflammatory response	Ccl24	NM_019577	Chemokine (C-C motif) ligand 24	-1.83	0.39	-0.08	-3.13
Leukotriene metabolic process	Ltc4	NM_008521	Mus musculus leukotriene C4 synthase	-0.30	0.37	-0.10	-3.01
Transcription	Rorb	AK044421	RAR-related orphan receptor beta	0.88	0.91	0.83	-2.91
Endocytosis	Mrc1	NM_008625	Mannose receptor, C type 1	-1.34	-0.42	-1.41	-2.81
Signal transduction	Calcr	NM_007588	Calcitonin receptor	-3.72	0.03	-2.71	-2.80
Leukotriene metabolic process	Alox12	NM_007440	Arachidonate 12-lipoxygenase	-0.73	0.35	-0.10	-2.72
NC	Msr2	NM_030707	Macrophage scavenger receptor 2	0.29	0.33	0.44	-2.59
Transport	Trpa1	NM_177781	Transient receptor potential cation channel	0.11	-2.00	-0.19	-2.52
Microtubule-based process	Kif1b	NM_207682	Kinesin family member 1B	-0.92	-1.69	-2.30	-2.48
Immune response	Cxcl4	NM_019932	Chemokine (C-X-C motif) ligand 4	-0.61	0.06	-0.52	-2.33
Translation	Eif2s3x	NM_012010	Eukaryotic translation initiation factor	-0.28	-2.47	-2.29	-2.27
Transcription	Zfp101	NM_009542	Zinc finger protein 101	-0.48	-0.36	-2.10	-2.20
Transcription	Irx6	NM_022428	Iroquois related homeobox 6	-1.12	-0.58	-1.20	-2.19
Prostaglandin biosynthesis	Ptgis	NM_008968	Prostaglandin I2	-0.67	-0.46	-1.19	-2.19
MAPK activity	Prok2	NM_015768	Prokineticin 2	-1.67	-0.53	-1.76	-2.13
Cell adhesion	Pcdh12	NM_017378	Protocadherin 12	-0.17	-0.27	-0.68	-2.10
Apoptosis	Ngfr	NM_033217	Nerve growth factor receptor	-1.74	0.32	-1.73	-2.00

NC Not classified

Genes were considered significantly upregulated or downregulated if the change in their relative expression levels was ≥ 2 fold or ≤ -2 fold, respectively.

### Validation of microarray data by real-time qRT-PCR analysis

**Figure 4:**
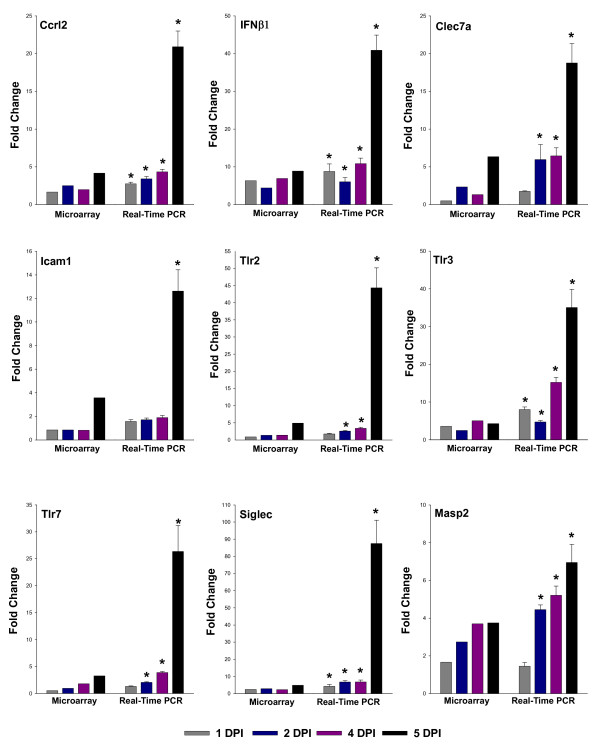
**Validation of Microarray data of selected genes by qReal-Time RT-PCR**. The mRNA expression levels of various genes were determined to validate the expression data of microarray analysis. The microarray and Real-Time PCR data is plotted in graph for Ccrl2, Ifnβ1, Clec7a, Icam1, Tlr2, Tlr3, Tlr7, Siglec and Masp2 respectively. Gapdh was used as housekeeping gene. The values are Mean ± SE of three biological replicates without pooling RNA. *Significantly different from control (mock-infected) mouse at p < 0. 05 by Dunnet's test.

To validate the differential gene expression profiles obtained by microarray analysis, expression of various receptors involved in immune response (Clec7a, Masp2), inflammatory response (Tlr2, Siglec1, Tlr7, and Ifnβ1), signal transduction (Ccrl2), cell adhesion (Icam1) and defense response (Tlr3) were examined by qRT-PCR. These genes were chosen on the basis of their significant regulation at early phase of infection or at disease severity phase. The data demonstrate that the overall results of qRT-PCR were consistent with those of the microarray analysis. Although several fold differences was observed between these two types of analysis because of intrinsic differences between the techniques, the RT-PCR results reveal the same relative regulation of transcription as the microarray data, and confirm that expression of the selected genes was significantly increased in response to infection with JEV (Figure 4).

## Discussion

The clinical outcome of a viral infection is largely dependent on the balance between host response and viral replication rates. The interaction of the virus with cell and evasion of the host immune response is crucial to the development of disease in a susceptible host. Japanese encephalitis (JE) is, at present, the single most important cause of viral encephalitis in Asia. In addition to causing acute illness with a high mortality rate, the disease may leave survivors with major mental and physical disabilities. Despite the fact that Japanese encephalitis is a major disease affecting the tropical world, little is known of its pathogenesis due, partly, to the lack of a suitable animal model and the complex cell interactions in infected individuals. Understanding the events that occur within the central nervous system after viral exposure is necessary if effective therapeutic interventions against viral encephalitis are to be developed.

The current investigation shows global transcription profile in the brain of JEV-infected animal in a natural route of exposure. In this study, after subcutaneous infection, animals started showing JE specific symptoms from 3-day post-infection. The tremendous increase in viral load was observed from 4 DPI in this animal model, which may be because of the completion of virus dissemination and release from brain cells. In addition to this, another mechanism behind this robust increase in viral load may be the increase in viral spread through a haematogenous route via endothelium [9], because at this time point of infection there was an increase in BBB permeability also. However, the detailed study is required to explore the possible mechanism of JEV entry in to the CNS. The greatest difference in the gene expression was observed when the viral load peaked in the brain. However, viral titre remains constant after 5 day until time to death; this confirms the role of host response in the severity of disease rather than virus itself. As reported earlier the pooled RNA samples were used for microarray analysis to reduce the effects of biological variation and to easily find the substantive differences [11]. Genes that were differentially expressed during infection can potentially provide insights into the complex regulatory phenomena in response to JEV infection.

The global transcription profile of the mouse brain infected with JEV revealed the activation of a variety of antiviral host defenses early after infection: activation of innate immune responses, protein processing, interferons and interferon-stimulated genes, complement system, activation of natural killer cells, macrophages and leukocyte infiltration into brain. These indicated the induction of an important local inflammatory response by the host after the infection of CNS. The major set of genes/pathways with increased expression observed in present study with JEV are similar to those reported for other neuroinvasive viruses like West Nile virus (WNV). These results suggest that some pathways are commonly activated during neurotropic viral infection of the CNS, and the gene products like Ifn-γ, Cxcl10/IP-10 etc. involved in protective roles at early phase of infection may also contribute to the pathogenesis of the disease at later stage [2,12].

In Flaviviral encephalitis both macrophage and T-cell infiltration appear to play an important role in the virus entry to CNS [13]. The extravasations of these inflammatory cells and generation of host response may require the activation of different mediators involving, respectively, selectins and their ligands, chemokines and chemokine receptors, proinflammatory cytokines, integrins and cell adhesion molecules (CAMs) and matrix metalloproteinases (MMPs).

Chemokines are now recognized as critical regulators of leukocyte trafficking into the CNS. Numerous studies have revealed that resident cell populations of the CNS are able to synthesize and secrete a variety of chemokines. Astrocytes and microglia are the primary source of chemokines following infection with a wide range of neurotropic viruses. Neurons are also capable of secreting chemokines during JEV and WNV infection [7,14]. The early increased expression of cytokine Tnf-α and T-cell activation chemokines, such as Cxcl10/IP-10, Cxcl11/I-TAC, and Cxcl9/Mig-1 suggests their role in the infiltration of leucocytes at an early phase of host response to highly neuroinvasive JEV infection. These chemokines were also up regulated in the brain of animals infected with highly neuroinvasive strains of WNV [12]. Various reports suggest that the interferon and interferon-regulated genes are playing important role in the activation of leukocytes and their infiltration to CNS. In this study, the earliest consistent transcriptional response was an increase in transcript cluster of Ifn-associated genes. This cluster includes Ifn-α, Ifn-β, and Ifn-γ regulated genes. These genes were also found to be involved in the pathogenesis of West Nile virus encephalitis [15,16].

Interestingly, chemokines which are responsible for infiltration of monocytes and macrophage like Ccl5/RANTES and Ccl4/MIP-1β showed increased expression at an acute phase of infection and increases the infiltration of monocyte and macrophages. At this phase of infection before severity of disease, there was an increase in expression of various proinflammatory mediators also like Tnf-α, Il1α, Il1β, Il-12 and Ccl2/MIP-1α along with chemoattractants. These immune mediators may be exacerbating the infiltration of inflammatory cells by increasing the expression of various integrins, cell adhesion molecules, selectins and increased blood-brain barrier permeability. A study with neural stem/progenitor cells infected with JEV also suggested the possible role of these cytokines in the cellular infiltration [17]. Thus, according to the present data it can be hypothesized that the uncontrolled expression of these mediators may be detrimental leading to the disease severity.

Cell-cell adhesion mediated by various genes is critical for interaction of lymphocytes and antigen presenting cells with endothelium and recruitment to the inflammation site. Recent report suggests that Icam1 plays an important role in WNV neuroinvasion and this is also involved in the blood-brain barrier permeability and the progression of neuroinflammation [18-20]. The combination of IFN-γ with TNF-α or IL-1 can strikingly up regulate the expression of these adhesion molecules [21] and our results also confirm the similar induction of these genes. Though adhesion molecules showed important role in neuroinflammation, a more thorough understanding will help to develop effective anti-inflammation strategies. The reports also suggested the importance of integrins in virus entry and a prominent endothelial cell receptor was also identified as the functional receptor for WNV and JEV in vertebrate cells [22].

Toll-like receptors signal transduction leads to the expression of several proteins that have important roles in the inflammation and immune response to virus. Recent report suggests that Tlr3 is involved in the WNV replication in brain and induction of neuronal injury, may be through inflammation induced cell death in the brain [23]. The present data also showed the increased expression of Tlr3 along with TNF-α at an early phase of JEV infection and it may be involved in virus entry and resultant encephalitis. Studies showed that the induction of Tlr2-mediated cytokine response in the brain contributes to the death of the animal [24]. Tlr2 and Tlr3 cooperation leads to the expression of the macrophage chemoattractants Ccl2 and Ccl5 [25], which may be the case in JEV infection also. Other receptors like Tlr1, Tlr4 and Tlr7 are known to be involved in the exacerbation of virus-induced inflammation and defense response [26,27]. The TLR mediated pathway has been supported with various other important signaling receptors like Lilrb3, Ptprc [28,29]. The transcription profile of these receptors during disease course suggest a cumulative effect of these genes in JEV pathogenesis.

The innate immune response of multicellular organisms is initiated by the binding of soluble and membrane-bound host molecules including lectins to the surface of pathogenic organism. These pattern recognition receptors (PRRs) are required for signal transduction during host response. Lectin receptors like dendritic cell receptor Clec7a, Clec4e are playing important role in the activation of macrophage and innate immune response [30,31]. Recent reports suggest the critical role of macrophage receptor Clec5a in dengue and Japanese encephalitis severity [8,32]. Other macrophage receptors like Masp2 and Siglec1 plays important role in inflammation [33,34]. Taken together, it can be hypothesized that, the JEV activates signal transduction through these receptors, exacerbates the inflammation, and disease severity. These are the newly explored pattern-recognizing C-type lectin receptors on dendritic cells and macrophages and targeting these molecules may provide improved therapeutic options.

The data also presents an interesting set of genes in JEV pathogenesis. These are the caspases, which are not only playing an essential role during apoptotic cell death, but a subfamily of them--the inflammatory caspase, are associated with immune responses to microbial pathogens [35]. These include caspase-1, 4, 5, 11 and 12. Activation of inflammatory caspases, such as caspase-1 and caspase-5, occurs upon assembly of an intracellular complex, designated the inflammasome [36]. The activation of various pattern recognition receptors like Tlr, C-type lectins and up regulation of Casapse-1, Caspase-4, Pycard, Cathepsin, Il1β and Il18bp indicates the possible generation of inflammasomes during JEV infection. The critical role of Cryopyrin/Nalp3 inflammasomes in case of virus infection has already been discussed [37]. These inflammasomes may be playing important role in JEV pathogenesis and further research is required to identify the role of these inflammasomes in JEV infection.

The present study also provides various important mediators of host anti-viral response at an early phase of infection, those with increased expression included Mx1 and Mx2 (myxovirus resistance 1 and 2), antiviral GTPases, as well as various members of the Oas family, Guanylate nucleotide binding proteins and TRIM protein family. Oas has shown involvement in west-nile virus infection also [38]. Some of these genes like, Oasl2, Oas1a, Oasl1, G1p2, Ifit3 and Iigp2 showed significant regulation at an early phase of infection and these results were consistent with our earlier report on host response in neuronal cell infected with JEV [7]. The significant regulation of these genes along with Trim proteins like Trim 30, 25 and Trim 34 was also observed in the brain of animals infected with highly neuroinvasive strains of WNV [12]. The genes of complement system like C1r, C2, C3, and C4b are known to recognize pathogen-associated molecular patterns (PAMPs) and their involvement in WNV infection is also reported recently [39]. Various hematopoietic cell surface molecules had increased expression, including some Ly6 (lymphocyte) antigens, a group of molecules that are involved in signal transduction and cell activation [40].

These data illustrate that the activation of various PRRs, provides a first line of defense by inducing interferons, proinflammatory cytokines and chemokines [41,42]. These mediators can promote tissue damage if not dampened in a timely manner, such as the increased leukocytic infiltration, which has aggravated the CNS inflammation and causes fatal encephalomyelitis during JEV infection. Recent studies also supported this notion of immunopathogenesis in JEV infection [43,44]. The role of chemokines can not be ignored in this neuroinflammation as they are critical mediators of neuropathology during JEV infection either by attracting pathogenic inflammatory cells or directly mediating neurotoxicity and cell death. The involvement of this aggravated inflammation in disease severity has already been studied with other viral diseases [45-47].

## Conclusion

In conclusion, there is clear evidence for an immunopathological mechanism in the pathogenesis of Japanese encephalitis in mice and may be of use in determining a role for anti-inflammatory agents in the disease management. One of the most interesting aspects of our results is the information on various receptors that may be involved in the complex interaction between host and virus. Resident brain cells appeared to be the source of early immune mediators while infiltrating leukocyte are playing important role in the severity of the disease. This will aid further attempts to control the inflammatory conditions during JEV infection. The further elucidation of significantly regulated receptor-ligand interaction and resulted signal transduction processes may demonstrate the complexity of the interplay between the virus and the host, and may open new ways for therapeutic strategies for diseases which has inflammation as the major cause of disease severity.

## Materials and methods

### Virus

The JaOArS982 strain of Japanese encephalitis virus was employed throughout this study. The virus was propagated in suckling BALB/c mice. The brain tissue was harvested when clinical signs of sickness became apparent. A 10% suspension of the brain tissue was made by homogenization in the minimum essential medium (MEM). It was then centrifuged at 10,000 × g to remove cellular debris and filtered through 0.22 μm sterile filters. The mouse brain tissue-derived virus was stored at -70°C in small aliquots and was used as the source of virus for all the experiments.

### Virus Titration

The infectivity of the virus stock (PFU JEV/ml) was assessed by a quantitative plaque-forming assay on the monolayers of porcine stable (PS) kidney cells. Monolayers of cells were inoculated with 10-fold dilutions of virus sample made in MEM containing 1% FBS and incubated for 1 h at 37°C with occasional shaking. The inoculum was removed by aspiration and the monolayers were further overlaid with 1.25% methylcellulose containing MEM with 1% FBS. After incubation for 4 days the overlay medium was removed, the cells were fixed with methanol and stained with 0.5% crystal violet, and the end-point titre was determined by macroscopic counting of plaques.

### Virus infection

We have adopted a new mice animal model for Japanese encephalitis virus infection with modification in age and the route of infection, as reported by us earlier [8]. Briefly, one-week-old BALB/c mice of either sex were injected subcutaneously with approximately 10^3 ^PFU (in 50 μl of PBS) of JEV. Control animals received the same volume of PBS as the experimental group. All experiments were performed according to the protocol approved by the Institutional Animal Ethics Committee.

### Viral load in the CNS of infected mice

Mice were sacrificed each day post-infection (DPI) from day 1 to day 6 to harvest brain tissue. The animals were perfused with cold PBS before harvesting the brain tissue. The cerebral cortex of brain tissue was homogenized in the minimum essential medium (MEM). It was then centrifuged at 10,000 × g and filtered through 0.22 μm sterile filters. Presence and growth kinetics of virus was confirmed and titrated in the prepared sample by plaque formation on the monolayers of porcine stable (PS) kidney cells.

### Histopathological Analysis

Mock- and JEV-infected animals were sacrificed after specific time points. Tissue sample of brain was dissected out and fixed in Bouin's solution. After fixation, small pieces were processed by automated tissue processor (Leica TP1020) dehydrated and embedded in paraffin wax. Multiple sections of 12-μm thickness were prepared using automatic microtome (Microm HM360) and stained with hematoxylin and eosin in Leica Autostain-XL. Microscopic observation was performed on sections of cerebral cortex under LEICA DMLB microscope and photographs were taken using Leica DC 500 camera.

### Sample Acquisition, RNA Isolation and Quality Control

Brain tissues were collected from mock- and JEV-infected group of three mice at different days post infection. The sections of cerebral cortex of brain tissue were stored in RNA *later *(Qiagen, Hilden, Germany) at -70°C until processed for RNA extraction. Total RNA was extracted using the Qiagen (GmbH, Hilden) RNAEasy Mini kit according to the instructions of the manufacturer. RNA quality and integrity was assessed using RNA 6000 Nano Lab Chip on the 2100 Bioanalyzer (Agilent, Germany) following the manufacturer's protocol. RNA samples with RIN (RNA Integrity Number) ≥ 8 were used in all experiments. Equal concentration of total RNA from three animals of each mock- and JEV-infected group were pooled and used for microarray analysis. Validation of microarray data of relevant genes was carried out by qRT-PCR with three biological replicates without pooling of RNA.

### Microarrays and Hybridization

Low RNA Input Fluorescent Linear Amplification Kit (Agilent, Santa Clara, CA) was used for labeling. Briefly, both first and second strand cDNA were synthesized by incubating 500 ng of pooled total RNA with 1.2 μl of oligo dT-T7 promoter primer in nuclease-free water at 65°C for 10 min followed by incubation with 4.0 μl of 5× First strand buffer, 2 μl of 0.1 M DTT, 1 μl of 10 mM dNTP mix, 1 μl of 200 U/μl MMLV-RT, and 0.5 μl of 40 U/μl RNaseOUT, at 40 °C for 2 h. Immediately following cDNA synthesis, the reaction mixture was incubated with 2.4 μl of 10 mM Cyanine-3-CTP or 2.4 μl of 10 mM Cyanine-5-CTP (Perkin-Elmer, Boston, MA), 20 μl of 4× Transcription buffer, 8 μl of NTP mixture, 6 μl of 0.1 M DTT, 0.5 μl of RNaseOUT, 0.6 μl of inorganic pyrophosphatase, 0.8 μl of T7 RNA polymerase, and 15.3 μl of nuclease-free water at 40 °C for 2 h. Qiagen's RNeasy mini spin columns were used for purifying amplified cRNA samples. The quantity and specific activity of cRNA was determined by using NanoDrop ND-1000 UV-VIS Spectrophotometer version 3.2.1. Samples with specific activity >8 were used for hybridization. 825 ng of each Cyanine 3 or Cyanine 5 labeled cRNA in a volume of 41.8 μl were combined with 11 μl of 10× Blocking agent and 2.2 μl of 25× Fragmentation buffer (Agilent), and incubated at 60°C for 30 minutes in dark. The fragmented cRNA were mixed with 55 μl of 2× hybridization buffer (Agilent). About 110 μl of the resulting mixture was applied to the Agilent Whole Genome Mouse 4 × 44 k Gene Expression Microarray (AMADID: 14868, Agilent Technologies) and hybridized in a two-color comparative format at 65°C for 17 h in an Agilent Microarray Hybridization Chamber (SureHyb: G2534A) with hybridization oven. After hybridization, slides were washed with Agilent Gene expression wash buffer I for 1 min at room temperature followed by a 1 min wash with Agilent gene expression wash buffer II for 37°C. Slides were finally rinsed with acetonitrile for cleaning up and drying. Hybridized arrays were scanned at 5 μm resolution on an Agilent DNA Microarray Scanner, Model G2565BA. Data extraction from images was done using Feature Extraction software of Agilent.

### Microarray Data Analysis

Feature extracted data was analyzed using GeneSpring Gx v 11.0 software from Agilent. Normalization of the data was done using per spot per chip intensity dependent lowess normalization. Further quality control of normalized data was done using correlation based condition tree to eliminate experimental error. Genes that had ≥2 (Up regulated) and ≤-2 (Down regulated) fold change at 5 DPI were filtered from the data, irrespective of their regulation at early time points and selected for further analysis. Differentially regulated genes were clustered using gene tree to identify significant gene expression patterns. Ontology based biological analysis was done using Gene Ontology browser in GeneSpring Gx.

### Real Time qRT-PCR

The quantitative real-time RT-PCR was carried out to validate the microarray data with three biological replicates without pooling of RNA, using gene-specific primers from Quanti Tect primer assay kit (Qiagen Germany). Quanti Fast one-step RT-PCR kit (Qiagen Germany) was used for real time PCR and Glyceraldehyde-3-phosphate dehydrogenase (Gapdh) was used as an endogenous reference gene [8]. The relative quantification level of expression was determined using the 2^nd ^derivative maximum analysis with the determination of the crossing points for each transcript. Crossing point values for each gene was normalized to the respective crossing point values for the reference gene Gapdh. Data are presented as normalized ratios of genes along with standard error using the Roche Applied Science E-method [48].

### Statistical analysis

For microarray, sequential Student's *t *test (time point versus mock) was used to identify genes differentially expressed (*P *≤ 0.05) for each group and the experiment was repeated once. The quantitative real-time RT-PCR data was analyzed by one-way ANOVA followed by Dunnet's test for comparison between mock- and JEV-infected groups. The level of significance was set at *P *≤ 0.05. The data were expressed as mean ± SE of three animals per group. The real-time RT-PCR experiments were repeated twice.

## Competing interests

The authors declare that they have no competing interests.

## Authors' contributions

NG conceived of the study, carried out experiments. NG and PVL analyzed results and drafted the manuscript. Both the authors read and approved the final manuscript.

## Supplementary Material

Additional file 1 **Figure S1. Histopathology of the brain of JEV infected mouse**. Hematoxylin/eosin-stained section of cerebral cortex of mice at different days post infection with subcutaneously infected JEV. (a) Control mouse brain showing normal arrangement of neurons in various layers with blood vessels and glial cells (b) Brain section showing congestion and dilation of blood vessel (thick black arrow) at 1 DPI (c) Brain section showing moderate dilation of blood vessel (thick black arrow) at 2 DPI (d) Brain section showing leukocyte infiltration (thin black arrow) at 4 DPI (e) Brain section showing perivascular cuffing (thick black arrow) accumulating leukocytes (thin black arrow) and neurodegenration at 5 DPI (f) Showing enlarged view of perivascular cuffing and leukocyte transmigration. (Scale bar = 50 μM). Click here for file

Additional file 2 **Table S1. Genes up regulated in mouse brain after infection with Japanese encephalitis virus, classified as being involved in immune response**. Genes were considered significantly upregulated or downregulated if the change in their relative expression levels was ≥ 2 fold or ≤ -2 fold, respectively. Click here for file

Additional file 3 **Table S2. Genes up regulated in mouse brain after infection with Japanese encephalitis virus, classified as being involved in inflammation**. Genes were considered significantly upregulated or downregulated if the change in their relative expression levels was ≥ 2 fold or ≤ -2 fold, respectively. Click here for file

Additional file 4 **Table S3. Genes up regulated in mouse brain after infection with Japanese encephalitis virus, classified as being involved in defense response**. Genes were considered significantly upregulated or downregulated if the change in their relative expression levels was ≥ 2 fold or ≤ -2 fold, respectively. Click here for file

Additional file 5 **Table S4. Genes up regulated in mouse brain after infection with Japanese encephalitis virus that can be involved in Interferon response**. Genes were considered significantly upregulated or downregulated if the change in their relative expression levels was ≥ 2 fold or ≤ -2 fold, respectively. Click here for file

Additional file 6 **Table S5. Genes up regulated in mouse brain after infection with Japanese encephalitis virus that can be classified as being involved in Proteolysis**. Genes were considered significantly upregulated or downregulated if the change in their relative expression levels was ≥ 2 fold or ≤ -2 fold, respectively. Click here for file

## References

[B1] Manger ID Relman DA How the host 'sees' pathogens: global gene expression responses to infection Curr Opin Immunol 2000 12 215 218 10.1016/S0952-7915(99)00077-1 10712949

[B2] Johnston C Jiang W Chu T Levine B Identification of genes involved in the host response to neurovirulent alphavirus infection J Virol 2001 75 10431 10445 10.1128/JVI.75.21.10431-10445.2001 11581411 PMC114617

[B3] Prosniak M Hooper DC Dietzschold B Koprowski H Effect of rabies virus infection on gene expression in mouse brain Proc Natl Acad Sci USA 2001 98 2758 2763 10.1073/pnas.051630298 11226313 PMC30212

[B4] Tsai TF New initiatives for the control of Japanese encephalitis by vaccination: minutes of a WHO/CVI meeting, Bangkok, Thailand, 13-15 October 1998 Vaccine 2000 18 Suppl 2 1 25 10.1016/S0264-410X(00)00037-2 10821969

[B5] Solomon T Ni H Beasley DW Ekkelenkamp M Cardosa MJ Barrett AD Origin and evolution of Japanese encephalitis virus in southeast Asia J Virol 2003 77 3091 3098 10.1128/JVI.77.5.3091-3098.2003 12584335 PMC149749

[B6] van den Hurk AF Ritchie SA Mackenzie JS Ecology and geographical expansion of Japanese encephalitis virus Annu Rev Entomol 2009 54 17 35 10.1146/annurev.ento.54.110807.090510 19067628

[B7] Gupta N Santhosh SR Babu JP Parida MM Rao PVL Chemokine profiling of Japanese encephalitis virus-infected mouse neuroblastoma cells by microarray and real-time RT-PCR: implication in neuropathogenesis Virus Res 2010 147 107 112 10.1016/j.virusres.2009.10.018 19896511 PMC7126115

[B8] Gupta N Lomash V Rao PVL Expression profile of Japanese encephalitis virus induced neuroinflammation and its implication in disease severity J Clin Virol 2010 49 4 10 10.1016/j.jcv.2010.06.009 20637688

[B9] German AC Myint KS Mai NT Pomeroy I Phu NH Tzartos J Winter P Collett J Farrar J Barrett A A preliminary neuropathological study of Japanese encephalitis in humans and a mouse model Trans R Soc Trop Med Hyg 2006 100 1135 1145 10.1016/j.trstmh.2006.02.008 16814333

[B10] Yasui K Neuropathogenesis of Japanese encephalitis virus J Neurovirol 2002 8 Suppl 2 112 114 10.1080/13550280290168019 12491161

[B11] Kendziorski C Irizarry RA Chen KS Haag JD Gould MN On the utility of pooling biological samples in microarray experiments Proc Natl Acad Sci USA 2005 102 4252 4257 10.1073/pnas.0500607102 15755808 PMC552978

[B12] Venter M Myers TG Wilson MA Kindt TJ Paweska JT Burt FJ Leman PA Swanepoel R Gene expression in mice infected with West Nile virus strains of different neurovirulence Virology 2005 342 119 140 10.1016/j.virol.2005.07.013 16125213

[B13] Chambers TJ Diamond MS Pathogenesis of flavivirus encephalitis Adv Virus Res 2003 60 273 342 full_text 14689697 10.1016/S0065-3527(03)60008-4 PMC7202458

[B14] Klein RS Lin E Zhang B Luster AD Tollett J Samuel MA Engle M Diamond MS Neuronal CXCL10 directs CD8+ T-cell recruitment and control of West Nile virus encephalitis J Virol 2005 79 11457 11466 10.1128/JVI.79.17.11457-11466.2005 16103196 PMC1193600

[B15] Guo JT Hayashi J Seeger C West Nile virus inhibits the signal transduction pathway of alpha interferon J Virol 2005 79 1343 1350 10.1128/JVI.79.3.1343-1350.2005 15650160 PMC544142

[B16] Morrey JD Day CW Julander JG Blatt LM Smee DF Sidwell RW Effect of interferon-alpha and interferon-inducers on West Nile virus in mouse and hamster animal models Antivir Chem Chemother 2004 15 101 109 15185728 10.1177/095632020401500202

[B17] Das S Ghosh D Basu A Japanese encephalitis virus induce immuno-competency in neural stem/progenitor cells PLoS One 2009 4 e8134 10.1371/journal.pone.0008134 19956550 PMC2780913

[B18] Dai J Wang P Bai F Town T Fikrig E Icam-1 participates in the entry of west nile virus into the central nervous system J Virol 2008 82 4164 4168 10.1128/JVI.02621-07 18256150 PMC2292986

[B19] Greenwood J Etienne-Manneville S Adamson P Couraud PO Lymphocyte migration into the central nervous system: implication of ICAM-1 signalling at the blood-brain barrier Vascul Pharmacol 2002 38 315 322 10.1016/S1537-1891(02)00199-4 12529926

[B20] Hubbard AK Rothlein R Intercellular adhesion molecule-1 (ICAM-1) expression and cell signaling cascades Free Radic Biol Med 2000 28 1379 1386 10.1016/S0891-5849(00)00223-9 10924857

[B21] Ren G Zhao X Zhang L Zhang J L'Huillier A Ling W Roberts AI Le AD Shi S Shao C Shi Y Inflammatory cytokine-induced intercellular adhesion molecule-1 and vascular cell adhesion molecule-1 in mesenchymal stem cells are critical for immunosuppression J Immunol 2010 184 2321 2328 10.4049/jimmunol.0902023 20130212 PMC2881946

[B22] Chu JJ Ng ML Interaction of West Nile virus with alpha v beta 3 integrin mediates virus entry into cells J Biol Chem 2004 279 54533 54541 10.1074/jbc.M410208200 15475343

[B23] Wang T Town T Alexopoulou L Anderson JF Fikrig E Flavell RA Toll-like receptor 3 mediates West Nile virus entry into the brain causing lethal encephalitis Nat Med 2004 10 1366 1373 10.1038/nm1140 15558055

[B24] Kurt-Jones EA Chan M Zhou S Wang J Reed G Bronson R Arnold MM Knipe DM Finberg RW Herpes simplex virus 1 interaction with Toll-like receptor 2 contributes to lethal encephalitis Proc Natl Acad Sci USA 2004 101 1315 1320 10.1073/pnas.0308057100 14739339 PMC337050

[B25] So EY Kim BS Theiler's virus infection induces TLR3-dependent upregulation of TLR2 critical for proinflammatory cytokine production Glia 2009 57 1216 1226 10.1002/glia.20843 19191335 PMC2706926

[B26] Thompson JM Iwasaki A Toll-like receptors regulation of viral infection and disease Adv Drug Deliv Rev 2008 60 786 794 10.1016/j.addr.2007.11.003 18280610 PMC2410298

[B27] McKimmie CS Johnson N Fooks AR Fazakerley JK Viruses selectively upregulate Toll-like receptors in the central nervous system Biochem Biophys Res Commun 2005 336 925 933 10.1016/j.bbrc.2005.08.209 16157304

[B28] Nakayama M Underhill DM Petersen TW Li B Kitamura T Takai T Aderem A Paired Ig-like receptors bind to bacteria and shape TLR-mediated cytokine production J Immunol 2007 178 4250 4259 17371981 10.4049/jimmunol.178.7.4250

[B29] Cross JL Kott K Miletic T Johnson P CD45 regulates TLR-induced proinflammatory cytokine and IFN-beta secretion in dendritic cells J Immunol 2008 180 8020 8029 18523265 10.4049/jimmunol.180.12.8020

[B30] Wells CA Salvage-Jones JA Li X Hitchens K Butcher S Murray RZ Beckhouse AG Lo YL Manzanero S Cobbold C The macrophage-inducible C-type lectin, mincle, is an essential component of the innate immune response to Candida albicans J Immunol 2008 180 7404 7413 18490740 10.4049/jimmunol.180.11.7404

[B31] Yadav M Schorey JS The beta-glucan receptor dectin-1 functions together with TLR2 to mediate macrophage activation by mycobacteria Blood 2006 108 3168 3175 10.1182/blood-2006-05-024406 16825490 PMC1895517

[B32] Chen ST Lin YL Huang MT Wu MF Cheng SC Lei HY Lee CK Chiou TW Wong CH Hsieh SL CLEC5A is critical for dengue-virus-induced lethal disease Nature 2008 453 672 676 10.1038/nature07013 18496526

[B33] Hartnell A Steel J Turley H Jones M Jackson DG Crocker PR Characterization of human sialoadhesin, a sialic acid binding receptor expressed by resident and inflammatory macrophage populations Blood 2001 97 288 296 10.1182/blood.V97.1.288 11133773

[B34] Iwaki D Kanno K Takahashi M Endo Y Lynch NJ Schwaeble WJ Matsushita M Okabe M Fujita T Small mannose-binding lectin-associated protein plays a regulatory role in the lectin complement pathway J Immunol 2006 177 8626 8632 17142762 10.4049/jimmunol.177.12.8626

[B35] Martinon F Tschopp J Inflammatory caspases and inflammasomes: master switches of inflammation Cell Death Differ 2007 14 10 22 10.1038/sj.cdd.4402038 16977329

[B36] Petrilli V Dostert C Muruve DA Tschopp J The inflammasome: a danger sensing complex triggering innate immunity Curr Opin Immunol 2007 19 615 622 10.1016/j.coi.2007.09.002 17977705

[B37] Kanneganti TD Body-Malapel M Amer A Park JH Whitfield J Franchi L Taraporewala ZF Miller D Patton JT Inohara N Nunez G Critical role for Cryopyrin/Nalp3 in activation of caspase-1 in response to viral infection and double-stranded RNA J Biol Chem 2006 281 36560 36568 10.1074/jbc.M607594200 17008311

[B38] Lucas M Mashimo T Frenkiel MP Simon-Chazottes D Montagutelli X Ceccaldi PE Guenet JL Despres P Infection of mouse neurones by West Nile virus is modulated by the interferon-inducible 2'-5' oligoadenylate synthetase 1b protein Immunol Cell Biol 2003 81 230 236 10.1046/j.1440-1711.2003.01166.x 12752688

[B39] Mehlhop E Diamond MS Protective immune responses against West Nile virus are primed by distinct complement activation pathways J Exp Med 2006 203 1371 1381 10.1084/jem.20052388 16651386 PMC2121216

[B40] Classon BJ Coverdale L Mouse stem cell antigen Sca-2 is a member of the Ly-6 family of cell surface proteins Proc Natl Acad Sci USA 1994 91 5296 5300 10.1073/pnas.91.12.5296 8202484 PMC43981

[B41] Paul S Ricour C Sommereyns C Sorgeloos F Michiels T Type I interferon response in the central nervous system Biochimie 2007 89 770 778 10.1016/j.biochi.2007.02.009 17408841

[B42] Pichlmair A Reis e Sousa C Innate recognition of viruses Immunity 2007 27 370 383 10.1016/j.immuni.2007.08.012 17892846

[B43] Biswas SM Kar S Singh R Chakraborty D Vipat V Raut CG Mishra AC Gore MM Ghosh D Immunomodulatory cytokines determine the outcome of Japanese encephalitis virus infection in mice J Med Virol 2010 82 304 310 10.1002/jmv.21688 20029807

[B44] Saxena V Mathur A Krishnani N Dhole TN An insufficient anti-inflammatory cytokine response in mouse brain is associated with increased tissue pathology and viral load during Japanese encephalitis virus infection Arch Virol 2008 153 283 292 10.1007/s00705-007-1098-7 18074098

[B45] Rowell JF Griffin DE Contribution of T cells to mortality in neurovirulent Sindbis virus encephalomyelitis J Neuroimmunol 2002 127 106 114 10.1016/S0165-5728(02)00108-X 12044981

[B46] Samuel MA Diamond MS Pathogenesis of West Nile Virus infection: a balance between virulence, innate and adaptive immunity, and viral evasion J Virol 2006 80 9349 9360 10.1128/JVI.01122-06 16973541 PMC1617273

[B47] Sugamata M Miyazawa M Mori S Spangrude GJ Ewalt LC Lodmell DL Paralysis of street rabies virus-infected mice is dependent on T lymphocytes J Virol 1992 66 1252 1260 1731103 10.1128/jvi.66.2.1252-1260.1992 PMC240838

[B48] Tellmann G Olivier G Light Cycler 480 Real-Time PCR System:Innovative Solutions for Relative Quantification Biochemica 2006 4 16 18

