# Supplementary material for: Transcriptomic profile of host response in Japanese encephalitis virus infection
Source: Virol J. 2011 Mar 4;8:92. doi: 10.1186/1743-422X-8-92 (PMC3058095; doi:10.1186/1743-422X-8-92)
Supplement: Additional file 1 — Figure S1. Histopathology of the brain of JEV infected mouse. Hematoxylin/eosin-stained section of cerebral cortex of mice at different days post infection with subcutaneously infected JEV. (a) Control mouse brain showing normal arrangement of neurons in various layers with blood vessels and glial cells (b) Brain section showing congestion and dilation of blood vessel (thick black arrow) at 1 DPI (c) Brain section showing moderate dilation of blood vessel (thick black arrow) at 2 DPI (d) Brain section showing leukocyte infiltration (thin black arrow) at 4 DPI (e) Brain section showing perivascular cuffing (thick black arrow) accumulating leukocytes (thin black arrow) and neurodegenration at 5 DPI (f) Showing enlarged view of perivascular cuffing and leukocyte transmigration. (Scale bar = 50 μM). [file 1743-422X-8-92-S1.PDF]

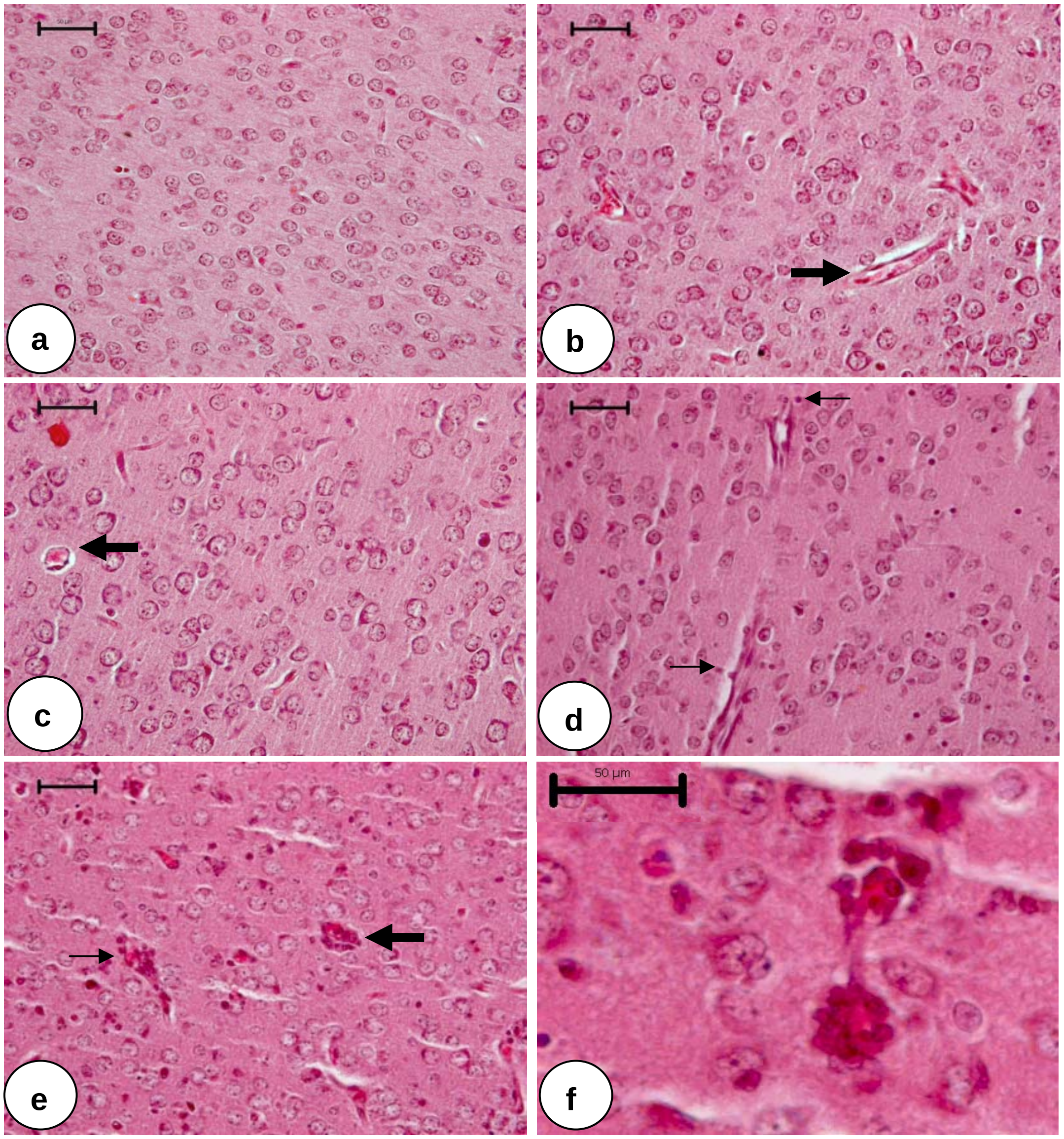

Figure S1 Histopathology of the brain of JEV infected mouse. Hematoxylin /eosin-stained section of cerebral cortex of mice at different days post infection with subcutaneously infected JEV. **(a)** Control mouse brain showing normal arrangement of neurons in various layers with blood vessels and glial cells **(b)** Brain section showing congestion and dilation of blood vessel (thick black arrow) at 1 DPI **(c)** Brain section showing moderate dilation of blood vessel (thick black arrow) at 2 DPI **(d)** Brain section showing leukocyte infiltration (thin black arrow) at 4 DPI **(e)** Brain section showing perivascular cuffing (thick black arrow) accumulating leukocytes (thin black arrow) and neurodegenration at 5 DPI **(f)** Showing enlarged view of perivascular cuffing and leukocyte transmigration. (Scale bar = 50 µM)
