# Supplementary material for: Transcriptomic profile of host response in Japanese encephalitis virus infection
Source: Virol J. 2011 Mar 4;8:92. doi: 10.1186/1743-422X-8-92 (PMC3058095; doi:10.1186/1743-422X-8-92)
Supplement: Additional file 2 — Table S1. Genes up regulated in mouse brain after infection with Japanese encephalitis virus, classified as being involved in immune response. Genes were considered significantly upregulated or downregulated if the change in their relative expression levels was ≥ 2 fold or ≤ -2 fold, respectively. [file 1743-422X-8-92-S2.PDF]

Table S1. Genes up regulated in mouse brain after infection with Japanese encephalitis virus, classified as being involved in immune response.

Accession No	Gene Symbol	Description	Fold change over mock-infected			
			1 DPI	2 DPI	4 DPI	5 DPI
**Oligoadenylate synthetase**						
NM_145153	Oas1f	2'-5' oligoadenylate synthetase 1F	4.89	4.22	5.28	6.05
NM_011854	Oasl2	2'-5' oligoadenylate synthetase-like 2	5.72	5.70	5.90	6.04
NM_145226	Oas3	2'-5' oligoadenylate synthetase 3	-1.87	2.06	3.29	5.83
NM_145227	Oas2	2'-5' oligoadenylate synthetase 2	4.72	4.07	4.61	5.34
NM_145209	Oasl1	2'-5' oligoadenylate synthetase-like 1	3.55	3.34	3.97	4.13
NM_145211	Oas1a	2'-5' oligoadenylate synthetase 1A	3.97	3.45	3.88	3.92
**Guanylate nucleotide binding protein**						
NM_153564	Gbp5	Guanylate nucleotide binding protein 5	3.28	2.19	4.17	6.70
NM_018734	Gbp3	Guanylate nucleotide binding protein 3	4.85	3.22	4.22	6.35
NM_010259	Gbp1	Guanylate nucleotide binding protein 1	2.33	1.63	1.1	5.34
NM_145545	Gbp6	Guanylate binding protein 6	3.88	2.30	4.27	5.10
NM_008620	Gbp4	Guanylate nucleotide binding protein 4	4.04	2.97	2.16	4.74
NM_010260	Gbp2	Guanylate nucleotide binding protein 2	3.79	2.62	3.90	4.66
**Other Genes**						
NM_010846	Mx1	Myxovirus resistance 1	6.20	3.85	6.94	7.76
NM_194336	Mpa2l	Macrophage activation 2 like	5.99	3.00	5.93	6.31
NM_013606	Mx2	Myxovirus resistance 2	5.06	3.58	5.24	6.22
NM_172603	Phf11	PHD finger protein 11	6.00	5.51	5.35	6.08
NM_199146	AI45161	Expressed sequence AI451617	4.07	2.68	3.96	6.06
NM_011579	Tgtp	T-cell specific gtpase	5.48	4.89	5.50	5.86
NM_172689	Ddx58	DEAD box polypeptide 58	3.73	2.08	3.98	4.90
NM_008880	Plscr2	Phospholipid scramblase 2	1.97	0.55	1.70	4.52
NM_009735	B2m	Beta-2 microglobulin	2.86	2.63	2.43	4.48
NM_181542	Slfn10	Schlafen 10	3.67	2.24	3.18	4.45
NM_013683	Tap1	Transporter 1,	3.97	2.11	3.43	4.32
NM_015790	Icosl	Icos ligand	1.21	0.53	1.61	3.92
NM_023158	Cxcl16	Chemokine (C-X-C motif) ligand 16	0.34	0.92	0.68	3.68
NM_018851	Samhd1	SAM domain and HD domain,	1.48	0.64	1.56	3.24
NM_011530	Tap2	Transporter 2,	2.16	1.23	2.14	3.23
NM_010821	Mpeg1	Macrophage expressed gene 1	1.96	1.71	2.62	3.22
NM_022964	Lat2	Linker for activation of T cells	0.75	1.32	0.64	2.51
NM_011691	Vav1	Vav 1 oncogene	0.57	0.86	0.62	2.28

Genes were considered significantly upregulated or downregulated if the change in their relative expression levels was ≥ 2 fold or ≤ -2 fold, respectively.
