# Supplementary material for: Transcriptomic profile of host response in Japanese encephalitis virus infection
Source: Virol J. 2011 Mar 4;8:92. doi: 10.1186/1743-422X-8-92 (PMC3058095; doi:10.1186/1743-422X-8-92)
Supplement: Additional file 3 — Table S2. Genes up regulated in mouse brain after infection with Japanese encephalitis virus, classified as being involved in inflammation. Genes were considered significantly upregulated or downregulated if the change in their relative expression levels was ≥ 2 fold or ≤ -2 fold, respectively. [file 1743-422X-8-92-S3.PDF]

Table S2. Genes up regulated in mouse brain after infection with Japanese encephalitis virus, classified as being involved in inflammation.

Accession No	Gene Symbol	Description	Fold change over mock-infected			
			1 DPI	2 DPI	4 DPI	5 DPI
NM_021274	Cxcl10	Chemokine (C-X-C Motif) Ligand 10	6.89	5.47	10.00	11.41
NM_013652	Ccl4	Chemokine (C-C Motif) Ligand 4	-0.60	0.93	3.97	8.48
NM_013653	Ccl5	Chemokine (C-C Motif) Ligand 5	0.76	1.61	4.23	6.77
NM_011331	Ccl12	Chemokine (C-C Motif) Ligand 12	3.81	2.55	5.42	6.26
NM_013654	Ccl7	Chemokine (C-C Motif) Ligand 7	2.76	2.75	2.79	6.02
NM_019494	Cxcl11	Chemokine (C-X-C Motif) Ligand 11	5.45	1.27	4.49	5.71
NM_008176	Cxcl1	Chemokine (C-X-C Motif) Ligand 1	3.92	3.02	3.99	5.36
NM_008987	Ptx3	Pentraxin related gene	0.45	-0.19	1.40	5.30
NM_019453	Mefv	Mediterranean fever	-0.85	0.13	-0.07	5.27
NM_011333	Ccl2	Chemokine (C-C Motif) Ligand 2	2.84	2.03	4.16	5.19
NM_018866	Cxcl13	Chemokine (C-X-C Motif) Ligand 13	3.70	2.90	3.89	5.15
NM_013693	Tnf	Tumor necrosis factor	3.65	2.83	3.83	5.06
NM_008599	Cxcl9	Chemokine (C-X-C Motif) Ligand 9	3.51	-0.57	2.52	5.06
NM_008352	Il12b	Interleukin 12b	-1.09	-0.61	-0.27	4.86
NM_008361	Il1b	Interleukin 1 beta	0.75	0.51	2.85	4.62
NM_031167	Il1rn	Interleukin 1 receptor antagonist	-0.91	-0.75	-1.30	4.46
NM_031168	Il6	Interleukin 6	-0.78	-0.73	1.74	4.33
NM_009263	Spp1	Secreted Phosphoprotein 1	0.45	0.44	0.69	4.00
NM_011337	Ccl3	Chemokine (C-C Motif) Ligand 3	-0.96	0.20	0.60	3.91
NM_010876	Ncf1	Neutrophil cytosolic factor 1	0.89	1.88	1.40	3.71
NM_010554	Il1a	Interleukin 1 alpha	-0.15	0.32	2.14	3.66
NM_008357	Il15	Interleukin 15 (Il15), mrna	-1.93	-1.57	1.09	3.33
NM_021443	Ccl8	Chemokine (C-C motif) ligand 8	0.79	0.13	-1.46	3.03
NM_012054	Aoah	Acyloxyacyl hydrolase	-0.89	-1.18	0.50	3.00
NM_011338	Ccl9	Chemokine (c-c motif) ligand 9	0.41	0.63	1.77	2.63
NM_011756	Zfp36	Zinc Finger Protein 36	-0.14	0.04	0.66	2.22

Genes were considered significantly upregulated or downregulated if the change in their relative expression levels was ≥ 2 fold or ≤ -2 fold, respectively.
