# Supplementary material for: Transcriptomic profile of host response in Japanese encephalitis virus infection
Source: Virol J. 2011 Mar 4;8:92. doi: 10.1186/1743-422X-8-92 (PMC3058095; doi:10.1186/1743-422X-8-92)
Supplement: Additional file 4 — Table S3. Genes up regulated in mouse brain after infection with Japanese encephalitis virus, classified as being involved in defense response. Genes were considered significantly upregulated or downregulated if the change in their relative expression levels was ≥ 2 fold or ≤ -2 fold, respectively. [file 1743-422X-8-92-S4.PDF]

Table S3. Genes up regulated in mouse brain after infection with Japanese encephalitis virus, classified as being involved in defense response.

Accession No	Gene Symbol	Description	Fold change over mock-infected			
			1 DPI	2 DPI	4 DPI	5 DPI
**MHC Class I presentation**						
NM_023124	H2-Q8	Histocompatibility 2, q region locus 8	4.26	4.13	3.17	5.41
NM_010398	H2-T23	Histocompatibility 2, t region locus 23	2.90	2.49	2.29	4.28
NM_001001892	H2-K1	Histocompatibility 2, k1, k region	2.64	2.57	1.86	3.76
NM_010394	H2-Q7	Histocompatibility 2, q region locus 7	2.55	2.39	1.76	3.73
NM_010391	H2-Q10	Histocompatibility 2, q region locus 10	2.55	2.63	1.76	3.68
NM_010392	H2-Q2	Histocompatibility 2, q region locus 2	2.37	2.36	1.81	3.65
NM_010380	H2-D1	Histocompatibility 2, d region locus 1	2.50	2.49	1.63	3.54
NM_010397	H2-T22	Histocompatibility 2, t region locus 22	2.07	2.24	1.09	3.27
NM_010390	H2-Q1	Histocompatibility 2, q region locus 1	2.35	2.15	1.57	3.13
NM_010378	H2-Aa	Histocompatibility 2, class ii antigen a,	1.00	-0.90	-1.01	3.06
NM_008199	H2-Bl	Histocompatibility 2, blastocyst	1.43	1.73	1.13	2.95
BC010322	H2-Ab1	Histocompatibility 2, class ii antigen a,	1.70	1.46	0.45	2.88
NM_010395	H2-T10	Histocompatibility 2, t region locus 10	2.75	1.88	0.94	2.82
NM_013819	H2-M3	Histocompatibility 2, m region locus 3	1.50	1.28	1.29	2.80
**Tripartite motif protein**						
NM_009277	Trim21	Tripartite motif protein 21	4.13	2.88	5.04	5.77
NM_009099	Trim30	Tripartite motif protein 30	3.68	3.06	3.78	4.65
NM_023835	Trim12	Tripartite motif protein 12	-4.91	0.90	1.82	4.23
NM_030684	Trim34	Tripartite motif protein 34	2.60	2.44	3.38	3.91
NM_201373	Trim56	Tripartite motif-containing 56	2.29	0.93	2.37	3.66
NM_009546	Trim25	Tripartite motif protein 25	2.89	2.20	2.59	3.50
**Lymphocyte associated GPI anchor binding Genes**						
NM_010738	Ly6a	Lymphocyte antigen 6 complex, locus A	3.34	2.14	3.28	4.26
NM_008879	Lcp1	Lymphocyte cytosolic protein 1	0.21	0.38	0.04	3.18
NM_020498	Ly6i	Lymphocyte antigen 6 complex, locus I	2.29	1.73	2.64	2.62
NM_008529	Ly6e	Lymphocyte antigen 6 complex, locus E	1.08	0.36	0.90	2.29
NM_008879	Lcp1	Lymphocyte cytosolic protein 1	0.73	0.93	0.20	2.20
NM_010741	Ly6c	Lymphocyte antigen 6 complex, locus C	1.66	0.60	2.00	2.16
NM_010696	Lcp2	Lymphocyte cytosolic protein 2	0.98	0.76	0.57	2.04

Genes were considered significantly upregulated or downregulated if the change in their relative expression levels was ≥ 2 fold or ≤ -2 fold, respectively.
