# Supplementary material for: Transcriptomic profile of host response in Japanese encephalitis virus infection
Source: Virol J. 2011 Mar 4;8:92. doi: 10.1186/1743-422X-8-92 (PMC3058095; doi:10.1186/1743-422X-8-92)
Supplement: Additional file 5 — Table S4. Genes up regulated in mouse brain after infection with Japanese encephalitis virus that can be involved in Interferon response. Genes were considered significantly upregulated or downregulated if the change in their relative expression levels was ≥ 2 fold or ≤ -2 fold, respectively. [file 1743-422X-8-92-S5.PDF]

Table S4. Genes up regulated in mouse brain after infection with Japanese encephalitis virus that can be involved in Interferon response.

Biological process	Gene Symbol	Accession No	Description	Fold change over mock-infected			
				1 DPI	2 DPI	4 DPI	5 DPI
Immune response	Ifit1	NM_008331	Interferon-induced protein	7.85	7.84	8.73	9.81
NC	Ifi44	NM_133871	Interferon-induced protein 44	7.60	4.56	7.84	9.63
Cytokine and chemokine signaling	Iigp1	NM_021792	Interferon inducible gtpase	8.01	4.55	7.43	9.24
Inflammation	Ifnb1	NM_010510	Interferon beta 1	6.34	4.41	6.94	8.89
Immune response	Ifi203	NM_008328	Interferon activated gene 203	1.77	-0.57	4.71	7.61
Immune response	Ifi202b	NM_011940	Interferon activated gene 202B	5.64	4.36	4.56	6.86
NC	Igtp	NM_018738	Interferon gamma induced gtpase	4.87	3.29	5.41	6.82
NC	Ifi205	NM_172648	Interferon activated gene 205	3.44	2.78	4.88	6.66
Immune response	Ifit2	NM_008332	Interferon-induced protein with tetratricopeptide	3.90	1.38	4.40	6.13
Transcription	Ifi204	NM_008329	Interferon activated gene 204	4.89	2.95	4.63	6.00
Response to virus	Ifi27	NM_029803	Interferon alpha-inducible protein 27	3.90	4.58	4.41	5.90
Immune response	Ifih1	NM_027835	Interferon induced with helicase C	2.46	2.07	4.61	5.93
Defense response	Ifi47	NM_008330	Interferon gamma inducible protein 47	5.83	2.8	4.52	5.74
Defense response	Ifna4	NM_010504	Interferon alpha 4	-0.82	-0.50	2.89	5.68
Transcription	Irf7	NM_016850	Interferon regulatory factor 7	5.31	5.41	5.15	5.58
Defense response	Ifnab	BC116864	Interferon alpha B	1.33	-0.03	2.03	5.38
NC	Iigp2	NM_019440	Interferon inducible gtpase 2	3.94	3.09	3.87	5.11
NC	Isg20	NM_020583	Interferon-stimulated protein	3.99	2.15	3.67	5.10
NC	Ifna12	NM_177361	Interferon alpha 12	-3.25	-0.26	3.91	5.04
Signal transduction	Stat1	NM_009283	Signal transducer and activator of transcription 1	4.49	3.46	4.20	4.75
Immune response	Ifi35	NM_027320	Interferon-induced protein 35	3.95	2.35	3.47	4.70
Defense response	Ifna2	K01411	Interferon alpha 2	-0.86	-0.56	2.76	4.29
Transcription	Irf1	NM_008390	Interferon regulatory factor 1	1.60	0.94	2.21	4.02
Response to biotic stimulus	Ifitm7	NM_028968	Interferon induced transmembrane protein 7	3.09	2.19	2.59	3.88
Transcription	Isgf3g	NM_008394	Interferon dependent positive acting transcription factor 3 gamma	3.59	2.94	3.60	3.87
Response to biotic stimulus	Ifitm6	NM_0010336	Interferon induced transmembrane protein 6	-0.24	-0.61	0.98	3.74
Inflammation	Ifng	NM_008337	Interferon gamma	-0.00	-0.49	0.20	3.57
NC	Gvin1	NM_029000	Gtpase, very large interferon inducible 1	2.09	1.09	2.27	3.32
Immune response	Ifit3	NM_010501	Interferon-induced protein with tetratricopeptide repeats 3	3.08	2.84	3.17	3.24
cell proliferation	Ifitm3	NM_025378	Interferon induced transmembrane protein 3	2.26	1.58	1.95	2.80
Transcription	Irf8	NM_008320	Interferon regulatory factor 8	0.90	1.21	1.35	2.74
Defense response	Ifna13	NM_177347	Interferon alpha 13	0.90	0.36	-0.00	2.65
Response to biotic stimulus	Ifitm1	NM_026820	Interferon induced transmembrane protein 1	0.46	0.39	0.08	2.45
Transcription	Irf5	NM_012057	Interferon regulatory factor 5	0.64	0.91	0.56	2.29
