# Supplementary material for: Transcriptomic profile of host response in Japanese encephalitis virus infection
Source: Virol J. 2011 Mar 4;8:92. doi: 10.1186/1743-422X-8-92 (PMC3058095; doi:10.1186/1743-422X-8-92)
Supplement: Additional file 6 — Table S5. Genes up regulated in mouse brain after infection with Japanese encephalitis virus that can be classified as being involved in Proteolysis. Genes were considered significantly upregulated or downregulated if the change in their relative expression levels was ≥ 2 fold or ≤ -2 fold, respectively. [file 1743-422X-8-92-S6.PDF]

Table S5. Genes up regulated in mouse brain after infection with Japanese encephalitis virus that can be classified as being involved in Proteolysis.

Accession No	Gene Symbol	Description	Fold change over mock-infected			
			1 DPI	2 DPI	4 DPI	5 DPI
**Complement Component**						
NM_023143	C1r	Complement component 1, r	3.81	0.61	2.53	4.98
NM_009778	C3	Complement component 3	-0.10	0.62	-0.34	4.13
NM_008198	Cfb	Complement factor B	3.65	3.78	3.60	3.98
NM_009780	C4b	Complement component 4B	2.01	2.25	1.39	3.64
NM_013484	C2	Complement component 2	1.62	1.24	1.34	3.60
NM_144938	C1s	Complement component 1, s	0.33	-0.43	1.45	2.80
NM_009777	C1qb	Complement component 1, q beta	1.21	1.28	1.03	2.67
NM_007572	C1qa	Complement component 1, q alpha	1.31	1.43	1.17	2.30
NM_007574	C1qc	Complement component 1, q C chain	1.20	1.37	0.98	2.25
**Other Genes**						
NM_007609	Casp4	Caspase 4	5.07	3.07	3.25	6.08
NM_017370	Hp	Haptoglobin	1.33	1.42	2.37	5.26
NM_010724	Psmb8	Proteosome subunit, beta type 8	3.98	3.11	3.67	5.17
NM_010370	Gzma	Granzyme A	-0.98	0.57	-0.10	5.05
NM_013585	Psmb9	Proteosome subunit, beta type 9	3.79	2.71	3.25	4.76
NM_009251	Serpina3g	Serine peptidase inhibitor	3.22	1.50	1.83	4.40
NM_021281	Ctss	Cathepsin s	1.98	2.79	2.42	4.01
NM_013542	Gzmb	Granzyme b	-0.60	1.04	0.34	3.97
NM_009982	Ctsc	Cathepsin c	0.66	1.05	0.60	3.52
NM_009807	Casp1	Caspase 1	1.51	0.58	1.75	3.38
NM_007972	F10	Coagulation factor X	1.86	1.03	0.89	3.37
NM_010809	Mmp3	Matrix metallopeptidase 3	-0.89	-0.60	-0.61	3.27
NM_023258	Pycard	PYD and CARD domain	1.51	1.09	1.53	3.02
NM_008607	Mmp13	Matrix metallopeptidase 13	1.46	-0.51	0.58	2.73
NM_009373	Tgm2	Transglutaminase 2	0.76	0.73	0.60	2.71
NM_009776	Serping1	Serine peptidase inhibitor clade G	0.25	0.42	-0.13	2.62
NM_007801	Ctsh	Cathepsin h	0.64	0.91	3.93	2.52
NM_008902	Pp11r	Placental protein 11 related	0.86	0.01	1.14	2.40
NM_011414	Slpi	Leukocyte peptidase inhibitor	1.24	0.25	2.56	2.28
NM_009256	Serpinb9	Serine peptidase inhibitor,member 9	1.38	0.27	1.27	2.27
NM_011454	Serpinb6b	Serine peptidase inhibitor,member 6b	1.34	0.29	1.62	2.20
NM_008611	Mmp8	Matrix metallopeptidase 8	-0.88	-1.04	-0.12	2.07
NM_013640	Psmb10	Proteasome subunit, beta type 10	1.21	0.21	1.00	2.06

Genes were considered significantly upregulated or downregulated if the change in their relative expression levels was ≥ 2 fold or ≤ -2 fold, respectively.
